# Admitted patients’ recall of their treating doctors’ names in adult wards of a South African district hospital

**DOI:** 10.4102/safp.v68i1.6257

**Published:** 2026-04-29

**Authors:** Emile H. Kotze, Elmar C. van Eeden, Shannon-Lee Rietkerk, Kelefile Moloantoa, Mpumelelo Ndaba, Roux Burger, Cornel van Rooyen, Johan Botes, Chantelle C. van der Bijl

**Affiliations:** 1 Department of Family Medicine, School of Clinical Medicine, Faculty of Health Sciences, University of the Free State, Bloemfontein, South Africa; 2 Department of Biostatistics, School of Biomedical Sciences, Faculty of Health Sciences, University of the Free State, Bloemfontein, South Africa; 3 Research and Development Unit, School of Clinical Medicine, Faculty of Health Sciences, University of the Free State, Bloemfontein, South Africa

**Keywords:** identification, name, doctor–patient communication, patient awareness, hospital wards

## Abstract

**Background:**

Best practice guidelines suggest that patients should be well acquainted with their healthcare provider, and that doctors should introduce themselves or be introduced by another member of the patient’s healthcare team. This aligns with the National Patients’ Rights Charter by the National Department of Health, which states that every patient has the right to be treated by a named healthcare provider. Nevertheless, many patients have trouble recalling the name of their doctor. This study aimed to determine whether patients admitted to the adult ward of the National District Hospital in the Free State province, South Africa, could recall their doctor’s name.

**Methods:**

Structured interviews were conducted with consenting patients to evaluate whether they knew their doctors’ names.

**Results:**

Only 10% of patients recalled their doctor’s name. Less than half reported that doctors introduced themselves, 41% noticed a name badge, 63% saw their doctor more than once daily and 86% understood the reason for admission. The education levels of the patients showed no discernible influence on the results.

**Conclusion:**

A minority of patients could recall their doctors’ names, indicating a potential gap in communication and connection. Many patients expressed uncertainty regarding their doctor’s name. This study underscores the importance of improving the visibility, accessibility and consistency of doctor identification methods in healthcare settings to strengthen the doctor–patient relationship and optimise patient care experiences.

**Contribution:**

This study underscores the necessity for institutions to prioritise and implement strategies to enhance patient–doctor recognition, thereby improving therapeutic relationships and potentially improving patient outcomes.

## Introduction

According to the National Patients’ Rights Charter by the National Department of Health, agreed to by the Health Professions Council of South Africa (HPCSA), every patient has the right to be treated by a named healthcare provider. Therefore, a patient has the right to know who is providing their healthcare and must be attended to by clearly identified providers.^1^ Yet, in the busy hospital environment with transient medical teams, it can be difficult for patients to remember their doctor’s name, even though best practices suggest that patients should become well acquainted with their doctors.^2,3,4^

Although limited data exist as to whether patients can recall their doctors’ names, available research suggests a significant disconnect in doctor–patient communication.^2^ One study found that 75% of inpatients could not recall any of their doctor’s names when asked who was in charge of their care.^4^ This lack of recognition can hinder the development of a positive therapeutic relationship, which is vital for patient satisfaction and effective care.^5,6,7^

The simple act of a doctor introducing themselves to patients, clearly identifying their name, title and role, can significantly mitigate this issue.^8^ In addition to fostering a sense of personal connection, this official introduction aids in addressing the inherent power imbalance that naturally exists in doctor–patient interactions.^5,6,7^ The importance of basic identification cannot be overemphasised, especially when the doctor–patient relationship is complicated by factors such as a lack of time, patient age and the complexity of multidisciplinary care.^7,9,10^

No studies directly examining patient recall of doctors’ names in South African or African hospitals were identified in recent literature. This absence highlights a critical knowledge gap in local contexts, where unique challenges like multilingual wards, high patient volumes and resource constraints in public facilities may exacerbate recall issues compared to international settings.

This study aimed to determine whether patients admitted to the adult wards of the National District Hospital (NDH) in the Free State Province, South Africa, could recall the names of their doctors, as a measure of adherence to basic patient–doctor communication standards.

## Research methods and design

### Study design

A cross-sectional design was chosen for this study using structured interviews to gather data from adult patients.

### Setting

The NDH in the Mangaung Metropolitan region, Free State, was chosen as the setting for this study. The region has a population of 787 803 served by three public hospitals. The NDH is part of the University of the Free State’s teaching platform. It has a maternity ward, four adult wards, a paediatric ward, a day ward for surgery, a casualty department and the Tshepong Crisis Centre that supports victims of domestic violence and sexual offences. It has a bed capacity of 150 beds managed by the Department of Family Medicine. Patients are either walk-in patients or referred from local clinics, private general practitioners, correctional services, the South African Police Service, as well as patients from surrounding Southern Free State towns. An estimated 10 patients who are 18 years and older are admitted from the casualty unit to the NDH wards daily. After the patients are admitted to the ward, only the dedicated ward doctor will consult them further, and they will not see their treating doctor from casualty again in the ward.

### Population, study sample and sampling

The study population consisted of all patients, 18 years and older, who had been admitted for at least one night to the NDH during the study period of September to November 2023. Consecutive sampling was done as all patients who met the inclusion criteria were included in the study. The estimated sample was approximately 450 patients per month. However, during the study, the sample was found to be approximately 100 patients per month. Hospital admissions rates could have fluctuated because of seasonal infection peaks, public holidays and changes in non-emergency presentations.

#### Inclusion criteria

The study included adult patients, 18 years or older, who were admitted to the adult wards at the NDH for at least one night, with admission before midnight, and included in the midnight bed statistics. Participants needed to be lucid and know their own name, location and the current month and year.

#### Exclusion criteria

Excluded patients included psychiatric patients who were under 72-h observation (as they are not capable of consent) or patients awaiting transfer, as well as any patient experiencing orientation difficulties. Those who were admitted for less than one night or day and not included in the midnight statistics were also excluded. For safety and logistical reasons, patients in isolation for infectious diseases, along with adult inpatients temporarily housed in the paediatric and maternity wards, were not included in this study.

### Measurement

The measuring tool was a structured interview form, uploaded to the data analysis software, REDCap (Research Electronic Data Capture), a secure web application for managing databases. The structured interview was developed after a literature review was conducted, and questions relevant to the study objectives were identified.^3,4,11,12^ Structured interviews were selected as the data-capturing tool to ensure consistent, comprehensive and comparable data collection across diverse patients. The structured interview form consisted of questions asking the participants their ward doctors’ names, which would answer the study’s main objectives as well as other subobjectives. Other questions explored what opportunities the patients had to learn their doctors’ names. For example, participants were asked whether their doctors introduced themselves or whether they were introduced by a third party (e.g. a nurse, another patient, etc.). Participants were also asked how often their doctors visited them to assess their familiarity with their doctors.

To explore factors influencing patient recall, the interview included a question regarding the patients’ preferences for formal or informal greetings. A formal greeting includes a polite salutation such as ‘good morning’, followed by the doctor’s full introduction (full name and title) and addressing the patient by name with appropriate titles. In contrast, an informal greeting typically uses casual phrases like ‘hi’ or ‘hello’, with the doctor introducing themselves by first name only and addressing the patient by name without titles. Information about the participants’ highest level of education was also asked. As part of the study’s objectives, participants were asked if they understood why they were hospitalised to confirm whether their doctor had explained their diagnosis in a way they could understand.

A set of screening questions included in the structured interview form determined whether participants were eligible for inclusion in the study. These questions were administered before the full structured interview began. The questions encompassed age, duration of admission, date and day of the week and location (checking for lucidity). All these questions were reflected in the inclusion criteria.

Interviews were conducted by trained researchers fluent in English, Afrikaans or Sesotho, supervised by a senior investigator.

### Pilot study

The pilot study was conducted at one of the wards at the NDH in Bloemfontein (information redacted to maintain the integrity of the review process after approval had been granted by the HSREC and the Free State Department of Health [FSDoH]). For the pilot, 20 patients who met the inclusion criteria were interviewed. The pilot study aimed to evaluate the effectiveness and appropriateness of the data collection method and tool in order to make the necessary adjustments before the main study started. No changes were made to the data collection tool, so the research continued as planned. The data from the pilot study were included in the main study as no changes were made to the finalised data collection method. The pilot study was conducted 1 week before the main study commenced.

### Data collection

After receiving approval from the Health Sciences Research Ethics Committee (HSREC) of the University of the Free State and the FSDoH, a pilot study was conducted, followed by the main study. For the main study, the researchers went to the hospital for 3 months, three times a week, for 1–2 h a day on Mondays, Wednesdays and Fridays. A courtesy letter was sent to inform the hospital’s clinical manager of the study. Logistical arrangements were made with each ward’s nursing unit manager. The researchers conducted a structured interview with participants using REDCap; the researchers asked the participants questions from the structured interview form and filled in their answers on REDCap.

Consenting participants signed a hard copy of the consent form (available in English, Afrikaans and Sesotho) prior to the structured interview. The consent form was available in English, Afrikaans and Sesotho, as per local demographics in the area. Interviews were conducted in the home language of the patient’s choice by researchers proficient in the language.

After each interview, the researchers reviewed patient files to verify the names of the treating doctors. This was also included in the structured interview form. The researchers focused on the doctors who wrote clinical notes and those who prescribed medication. The wards’ monthly rosters were also used to determine which doctors were allocated to each ward. After the participants were interviewed, the researchers put a red sticker on each patient’s file to ensure they did not duplicate interviews during the study period. The ward doctors were not aware of what the conducted interviews were about or what the stickers meant on the files.

Data were collected for the predetermined study period of 3 months, and 356 patients were interviewed per the inclusion criteria. This study period was selected because of the availability of researchers to attend out-patient department during their studies.

### Data management plan

Data were collected by the researchers who used the REDCap app on their mobile phones. All data were thus stored on the REDCap database. Only the researchers and the study leader had access to the stored data. Researchers had to log in to REDCap with a username and password to access the data. At the end of the data collection period, all data were sent to the Department of Biostatistics, University of the Free State for analysis.

### Data analysis

A data analyst from the Department of Biostatistics, University of the Free State assisted with the statistical analyses using SAS® (Statistical Analysis System, version 9.4).

Numerical variables were summarised by medians, minimums, maximums or means and standard deviations. Categorical variables were summarised by frequencies and percentages. The differences between groups were evaluated using appropriate statistical tests (chi-square and Fisher’s exact test) for unpaired data. A post-hoc power analysis was performed to verify that the sample size was sufficient to detect significant associations for the primary outcomes, using a conventional minimum power requirement of 0.80.

The total number of responses for individual questions varied slightly because of participants occasionally declining to answer or providing incomplete responses. Percentages were calculated based on the number of respondents for each specific item (*n*) rather than the total study population.

### Ethical considerations

Before the study and pilot study commenced, ethical clearance was obtained from the University of the Free State Health Sciences Research Ethics Committee (HSREC). The ethical clearance number is UFS-HSD2023/0345/2609. Approval for the study was also obtained from the Head of the Department of Family Medicine.

Ethical considerations included the right to privacy of personal information, voluntary participation, the right to withdraw without repercussion and implied consent. All data were stored on REDCap, which, as previously mentioned, is a secure web application for managing databases, which is behind the University of the Free State’s firewall. All participant information collected was anonymous and complied with the national *Protection of Personal Information Act*. Participants incurred no costs. Refusal to participate in the study had no detrimental effect on the participants, and appropriate precautions were taken to limit harm to participants. The right to access and control the collected data was limited exclusively to the members of the research team and the research supervisor. Finally, the participants were provided with the contact details of relevant authorities via the participant information sheet, should they have wanted to gain access to their responses or the final research findings in the future.

## Results

In total, there were 471 patients admitted across four wards at the NDH during the study period. However, of the 471 patients, 356 patients met the inclusion criteria and gave consent for participation in the study.

### Sociodemographic features

Table 1 summarises the sociodemographic profile of the study sample. The patient cohort exhibited a wide age range, which spans from 18 to 93 years; the median age was 48 years. The central 50% of the sample fell between the ages of 35 and 62 years. Female patients constituted a slight majority of respondents (*n* = 182; 51.3%).

**TABLE 1:** Sociodemographic profile of the patient sample.

Variable	*n*	%
**Sex (*n* = 355)**		
Female	182	51.3
Male	173	48.7
**Highest completed level of schooling (*n* = 355)**		
Grade 9 and below	124	34.9
Matric certificate	92	25.9
Grades 10–11	78	22.0
Tertiary	35	9.9
No schooling	26	7.3
**Method of admission (*n* = 354)**		
Emergency room	254	71.7
Elective	100	28.3

Most of the patients received at least some form of schooling. The patients reported Grade 9 and below most frequently as their highest level of education (*n* = 124; 35%), followed by a matriculation certificate (*n* = 92; 26%). Only 7.3% (*n* = 26) of the interviewed patients had no schooling at all.

A large proportion entered the hospital through the Casualty Department (*n* = 254; 71.8%), while the remaining 28.8% (*n* = 100) were admitted electively. Data were missing for two participants.

### Patient responses

As per Table 2, 87.1% (*n* = 310) of the participants did not know the name of their doctor, while only 10.1% (*n* = 36) did know their doctor’s name, and 2.8% (*n* = 10) were unsure. Of the 36 patients who knew their doctor’s name, 69.4% (*n* = 25) were male, and 30.6% (*n* = 11) were female. These results had a statistically significant chi-square *p*-value of 0.0031, which leads to the conclusion that male patients were more likely to know their doctor’s name than female patients.

**TABLE 2:** Patient responses on doctor name recall and identification methods.

Variable	*n*	%
**Do you know your ward doctor’s name? (*n* = 356)**		
No	310	87.1
Yes	36	2.8
Not sure	10	10.1
**Did the ward doctor introduce himself or herself? (*n* = 355)**		
Yes	170	47.9
No	153	43.1
Not sure	32	9.0
**If the doctor did not introduce themselves, did someone else introduce them? (*n* = 153)**		
No	138	92.6
Not sure	6	4.0
Yes	5	3.7
**How did the ward doctor greet you? (*n* = 350)**		
Formally	190	54.3
Informally	160	45.7
**Did your ward doctor wear a name badge? (*n* = 355)**		
Yes	147	41.4
No	145	40.8
Not sure	63	17.8
**How often did you see the ward doctor since being admitted to the ward? (*n* = 356)**		
Multiple time a day	224	62.9
Once a day	118	33.2
Not at all	14	3.9
**Do you understand why you were admitted to the hospital? (*n* = 355)**		
Yes	304	85.6
No	43	12.1
Not sure	8	2.3

A post-hoc power analysis was conducted to evaluate the strength of the association between gender and doctor name recall. With a sample size of 356 and an effect size of 11.55, the calculated statistical power was 0.864. This exceeds the conventional threshold of 0.80, suggesting the study was sufficiently powered to detect the observed association.

In response to the question ‘Did the doctor introduce themselves?’, less than half (*n* = 170; 47.9%) of patients indicated that their doctor introduced themselves, while 9% were unsure. These data are illustrated in Table 2. In response to the follow-up question, ‘If your doctor did not introduce themselves, did someone else introduce them?’, only 3.7% (*n* = 5) reported their doctor being introduced by someone else, while 92.6% (*n* = 138) said that no one else introduced their doctor.

**FIGURE 1:**
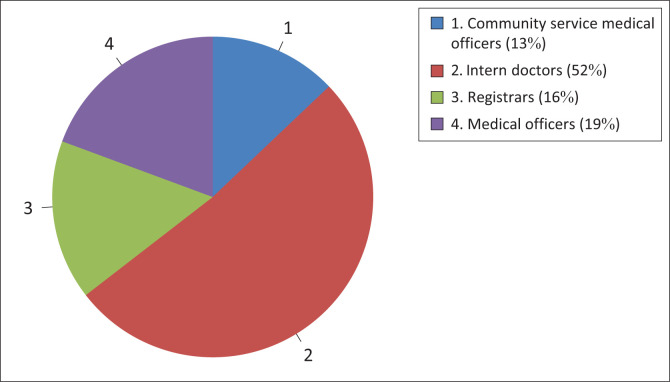
Categories of doctors whose names could be recalled.

A total of 36 patients could recall their doctors’ names. The categories of doctors whose names were recalled included 16 different intern doctors (forming the majority), six medical officers, five registrars and four community service medical officers (see Figure 1). When the participants were asked how they were greeted, 54.3% (*n* = 190) stated that they had received a formal introduction, while 45.7% (*n* = 160) indicated that they had received an informal greeting (as per the criteria stated previously). Of the 36 patients who remembered their doctor’s name, 50% (*n* = 18) had received an informal greeting, while the remaining 50% (*n* = 18) recalled receiving a formal greeting. There was no statistical significance found between the formality of the doctors’ introductions and the patients’ recollection of their names.

The participants were also asked whether their doctors had worn a name badge. In total, 41.4% (*n* = 147) indicated that their doctor had worn a name badge. Of that 41.4%, however, only 10% could actually recall their doctor’s name. Of the total sample, 40.9% (*n* = 145) of the patients indicated that their doctor did not wear a name badge, while 17.9% (*n* = 64) were unsure.

The overwhelming majority of patients (*n* = 224; 62.9%) saw their doctor more than once a day, followed by 33.2% (*n* = 118) of the patients, who saw their doctor once a day. Only 3.9% (*n* = 14) of the patients indicated they had not seen their doctor at all. The majority of the patients (*n* = 304; 85.6%) knew why they had been admitted, while 12.1% (*n* = 43) did not know at all, and 2.3% (*n* = 8) were unsure.

## Discussion

When doctors introduce themselves to their patients, they create a positive relationship, initiating patient–doctor communication that promotes full collaboration among doctors, patients and families.^8^ A simple introduction can ensure patient respect and dignity and also enhance information sharing that could help with diagnosing the patient.^7^ Additionally, the National Patients’ Rights Charter by the National Department of Health, as agreed to by the HPCSA, states that every patient has the right to be treated by a named health care provider.^1^ And yet, the results of this study show that only 47.9% of patients could confirm that their doctor had introduced themselves. It is also important to select the appropriate greeting for every situation and patient. This study found that 54.3% of doctors greeted their patients formally, although studies show that informal greetings are used more often, as most medical situations require a more relaxed tone.^5^ For example, a prospective survey performed on patients and caregivers at the Davidoff Cancer Institute in Israel found that patients preferred being greeted informally, as it creates a casual atmosphere, which is important in the healthcare environment as stressful environments can decrease memory.^12^ In a more relaxed environment, possibly created through informal greetings, patients may be more likely to remember their doctors’ names.^13^ Interestingly, however, the results of this study show that of the 36 patients who remembered their doctor’s names, 50% recalled being formally greeted by their doctors.

Equally significant is the data showing that only 10.1% of the interviewed patients knew their doctor’s name. These results are significantly lower than those obtained from similar studies completed in Australia and Saudi Arabia, which reported that 43% of patients knew their doctor’s name.^4,5^ In this study, 62.9% of the patients had seen their doctors multiple times a day. Although doctors are unlikely to introduce themselves more than once, repeated interactions provide patients with several opportunities to inquire about their doctor’s name. Notably, of the categories of doctors who could be named, 51.6% were interns, which might be because of them having recently graduated, and their training to introduce themselves to patients was still fresh in their minds. They might also have more time and fewer responsibilities than senior doctors. The other categories of more experienced doctors, namely community service doctors, registrars and medical officers, had likely forgotten this training or had perhaps thought their name badges would suffice. Although 41.4% of patients confirmed their doctor wore a name badge, only 10.1% recalled the name, with reasons possibly because of poor visibility or readability, patients not noticing it, difficult names or anxiety.

There are several factors that may influence why a patient does or does not remember their doctor’s name. In this study, for example, 71.8% of patients were admitted through the casualty department; they, therefore, may have had contact with several healthcare providers, which could have made remembering their primary doctor’s name difficult. Of the 36 patients who knew their doctor’s name, 69.4% were male. This statistically significant finding (*p* = 0.0031) echoes that of a Saudi Arabian study, which also showed that male patients were more aware of their doctor’s names and specialities.^4^ No statistical difference existed in name recall across education levels, including among the smallest group with no formal schooling.

### Study strengths and limitations

Several members of the research team were bilingual, and yet the researchers still found that language was a barrier to the data collection process. Practical constraints included navigating crowded, open wards with limited privacy. Patients admitted via casualty may have encountered multiple healthcare practitioners, potentially complicating name recall. Structured interviews were administered by trained multilingual researchers using a standardised REDCap form to minimise researcher bias and ensure consistent question delivery across participants. A key limitation is the inpatient ward setting at a busy district hospital, where high patient volumes, frequent staff rotations and acute illness likely represent suboptimal conditions for name recall and introductions – potentially underestimating performance in outpatient or less acute environments.

Some participants declined to answer specific questions or provided incomplete responses. The impact of these missing data on statistical power and the validity of the findings was minor, given the small number of missing data points.

### Recommendations

To improve patient–doctor relationships and increase patients’ awareness of their doctors’ names, hospitals should implement clear policies. Doctors should introduce themselves and wear visible forms of identification during every patient interaction. Doctors’ names should also be posted at the patient’s bedside. Giving patients business cards is also advised. All medical staff must receive communication skills training as part of these initiatives, and patient feedback is needed to track the efficacy of such training. Ultimately, a patient’s right to know their healthcare providers must be respected. Future studies should replicate this work in outpatient clinics and casualty departments to compare name recall across care settings, isolating the effects of acuity and visit frequency.

## Conclusion

This study aimed to determine whether patients admitted to the adult wards of the NDH in the Free State province knew their doctors’ names. The aim of the study was ultimately to assess the real-world adherence to basic patient–doctor communication standards. Only 10.1% of patients could remember the name of their doctor, which is below the percentages reported in international literature. Although the right to be treated by a named healthcare practitioner is protected under the National Patients’ Rights Charter, just 47.9% of patients said their doctor introduced themselves. Adherence to communication norms and ensuring that patients are informed about the identities of their care providers are essential for fostering patient–doctor relationships and protecting patient dignity.
